# Interactions between Fragmented Seagrass Canopies and the Local Hydrodynamics

**DOI:** 10.1371/journal.pone.0156264

**Published:** 2016-05-26

**Authors:** Nazha El Allaoui, Teresa Serra, Jordi Colomer, Marianna Soler, Xavier Casamitjana, Carolyn Oldham

**Affiliations:** 1 Department of Physics, University of Girona, 17071, Girona, Spain; 2 School of Civil, Environmental and Mining Engineering, The University of Western Australia, Perth WA 6009 Australia; Coastal Carolina University, UNITED STATES

## Abstract

The systematic creation of gaps within canopies results in fragmentation and the architecture of fragmented canopies differs substantially from non-fragmented canopies. Canopy fragmentation leads to spatial heterogeneity in hydrodynamics and therefore heterogeneity in the sheltering of canopy communities. Identifying the level of instability due to canopy fragmentation is important for canopies in coastal areas impacted by human activities and indeed, climate change. The gap orientation relative to the wave direction is expected to play an important role in determining wave attenuation and sheltering. Initially we investigated the effect of a single transversal gap within a canopy (i.e. a gap oriented perpendicular to the wave direction) on hydrodynamics, which was compared to fully vegetated canopies (i.e. no gaps) and also to bare sediment. The wave velocity increased with gap width for the two canopy densities studied (2.5% and 10% solid plant fraction) reaching wave velocities found over bare sediments. The turbulent kinetic energy (TKE) within the gap also increased, but was more attenuated by the adjacent vegetation than the wave velocity. As expected, denser canopies produced a greater attenuation of both the wave velocity and the turbulent kinetic energy within an adjacent gap, compared to sparse canopies. Using non-dimensional analysis and our experimental data, a parameterization for predicting TKE in a canopy gap was formulated, as a function of easily measured variables. Based on the experimental results, a fragmented canopy model was then developed to determine the overall mixing level in such canopies. The model revealed that canopies with large gaps present more mixing than canopies with small gaps despite having the same total gap area in the canopy. Furthermore, for the same total gap area, dense fragmented canopies provide more shelter than sparse fragmented canopies.

## Introduction

Vegetated systems cover less than 0.5% of the sea bed but account for up to 70% of the carbon storage in ocean sediments [1]. The potential for seagrass to store carbon is partially attributed to erosion protection and sediment stabilization afforded by seagrass meadows [2]. Therefore, as ecosystem engineers, aquatic plants play a critical role in protecting coastal areas however there are knowledge gaps surrounding the conditions that optimize this function [3]. The sediment stabilization is related to the absorption of kinetic energy by submerged coastal canopies, through the reduction of turbulence [4–7], waves [5,8–10], and mean currents [11,12]. Pujol et al. [5,7] showed that both flexibility and canopy density play a major role in the effectiveness of the canopy to attenuate waves and turbulence. Anderson and Smith [13] found that canopy density and the submergence ratio (defined as the ratio of plant to water height) were the main factors in determining wave attenuation. Paul and Amos [14] showed that canopies need a minimum shoot density to initiate wave attenuation, and Paul et al. [15] found that leaf length, canopy density and blade stiffness were crucial in determining the degree of wave attenuation. Furthermore, they found that the presence of a tidal current reduced considerably the effectiveness of wave attenuation by a canopy. Flow attenuation by canopies can also result in reduced sediment resuspension [11,16–19]. Seagrass canopies play an important role in particle trapping therefore increasing water transparency [20] and improving water quality. Seagrass meadows are found in many shallow coastal zones and support a large variety of infauna [18,21–23]. However they are constrained by light limitation [24] and benthic—wave interactions [25]. Therefore aquatic plants are recognized as both ecosystem engineers and water quality indicators, and their restoration and protection is a priority given that their regrowth is slow and variable [26,27].

Seagrass meadows are vulnerable to environmental changes, and are impacted by anthropogenic activities and climate change scenarios including increasing sea level. Low shoot densities, high mortality rates and high fragmentation (the patchiness of previously continuous habitats) are indicators of seagrass meadow degradation [28]. Patchiness, or gaps, within a canopy leave the bottom exposed to both waves and currents, and the underlying matte is no longer protected from erosion [1]. Different types of gaps within a canopy have been defined: erosive intermattes, sagittal channels, border intermattes and structural intermattes, and all types may have natural or anthropogenic origins [29,30]. Storms may erode mattes by tearing whole sections away, or by scouring sediments; these would correspond to erosive intermattes. Sagittal channels run perpendicular to the coast and are formed by return currents transporting wind- and/or wave-mixed surface waters to depth. Erosive intermattes appear as oval potholes in seagrass mattes and are likely formed by whirling currents carrying rocks and stones to depth, locally destroying the mattes [31]. The formation of gaps within meadows may also be triggered by human activities such as anchoring, trawling, fish farming, laying cables and pipes [32]. Gaps may also be defined according to whether they lie parallel (longitudinal) or perpendicular (transversal) to the dominant wave direction. Canopy fragmentation leads to habitats that are more vulnerable to external pressures than continuous canopies. Gera et al. [33] found that small patches of vegetation in fragmented canopies had lower canopy densities, shorter leaves and lower nutrient stores than continuous canopies, possibly an impact of stronger waves and currents experienced within the small patches compared to large ones.

While the fragmentation of seagrass canopies has been documented [1], few studies have investigated the effect of gaps on hydrodynamics. Folkard [34] found different flow regimes in unidirectional flows through transversal gaps in submerged vegetation and showed that the Reynolds number (based on over-canopy velocities and gap depth) and the gap aspect ratio were the most important parameters in determining the modification of bed shear stress. Koch and Gust [35] found that waves were able to penetrate more deeply within a canopy, due to the flexibility of the blades, compared to uni-directional flow. Greater penetration of wave energy induced increased mixing between the within-canopy and above-canopy waters. In one of the few studies on the effects of gaps on adjacent vegetation, El Allaoui et al. [36] found that longitudinal gaps in seagrass meadows had important effects on the flow within the nearby vegetation. They also found that lateral vegetation induced a sheltering inside the gap that was higher for dense canopies compared to sparse canopies. While longitudinal gaps might be more exposed to the ambient hydrodynamics, transversal gaps may remain sheltered by the nearby vegetation, which in turn implies that canopy ecology would respond differently to different gap orientations. It is also possible that fragmentation impacts vary according to gap orientation.

To provide direct comparisons with previous work on longitudinal gaps, we first investigated the hydrodynamics within a single transversal gap, the turbulent mixing within a fragmented canopy and the conditions under which adjacent vegetation shelters the gap. Attention was paid to the modification of ambient hydrodynamics as a function of the gap width and the canopy density. Laboratory experiments were conducted on a transversal gap in a canopy exposed to waves, simulating structural disturbances of shallow seagrass meadows that are dominated by waves. Finally, a simple fragmentation model generalized our experimental data across a theoretical canopy, to evaluate the canopy-averaged interactions between the gaps and turbulent mixing.

## Materials and Methods

**Fig 1:**
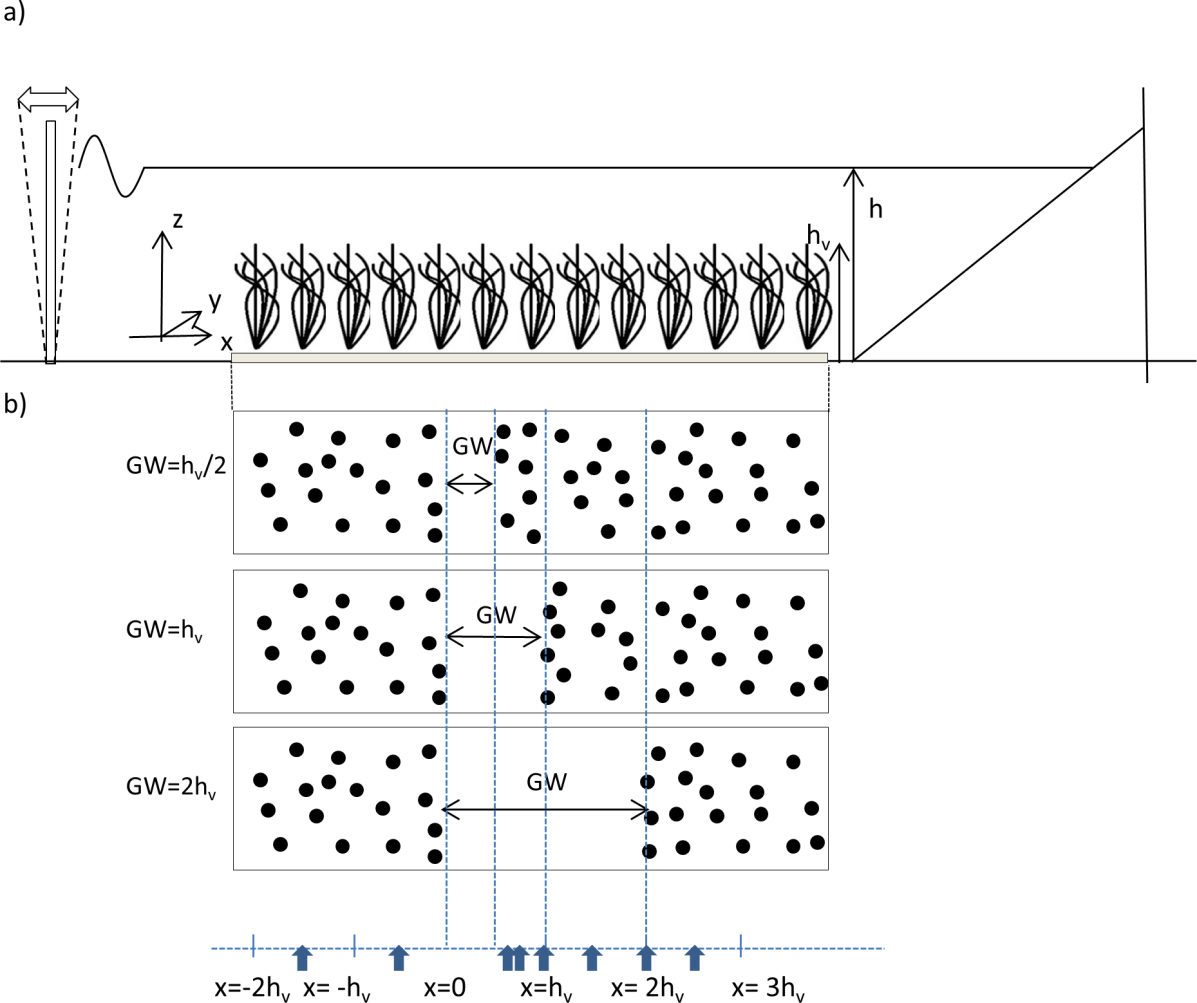
Schematic of the experimental set up. a) Side-view of the flume showing the canopy, the wave maker and the beach. b) Top-view of the three different gaps studied. h_v_ is the vegetation height, h is the height of the water column and GW is the gap width.

The research was carried out in a flume (6 m-long, 0.5 m-wide and 0.5 m-deep) where the mean water height, h, was 0.3 m (Fig 1A). The flume was equipped with a vertical paddle-type wave maker at the entrance. The vertical paddle was driven by a variable-speed motor that operated at a frequency of 1.2 Hz. This frequency was chosen to align with previous work [5,6,37,38], and it induced wavelengths of 1.03 m, corresponding to transitional water waves which are typical in regions dominated by aquatic vegetation. A plywood beach (slope 1:3) was placed at the end of the flume and covered in foam to better attenuate incoming waves. For details of the experimental set up see Pujol et al. [5]. We define the longitudinal direction, x, to be zero at the start of the gap; the lateral direction, y, is zero at the centerline of the tank, and the vertical direction, z, is zero at the flume bed (Fig 1B).

In this study we used a model of flexible vegetation with height h_v_ = 14 cm, constructed following the details of Pujol et al. [6]. The base of the flume was covered with 1-cm thick PVC boards perforated with holes 1 cm in diameter, in which the plants were placed. Plants consisted of polyethylene blades attached with a plastic band to a PVC dowel (2 cm long, 1 cm diameter). Empty holes in the PVC boards were filled with dowels (1 cm long, 1 cm diameter). The model plants were dynamically and geometrically similar to typical seagrasses [5,39].

The canopy density was determined as the Solid Plant Fraction (SPF). The SPF can be defined [7] as the fraction of the bottom boundary occupied by stems SPF(%) = n_stems_A_stem_/A_total_×100, where n_stems_ is the number of stems, A_stem_ is the horizontal area of each stem (A_stem_ = πd^2^/4), where d is the plant diameter and A_total_ is the total horizontal area occupied by the canopy. Two SPF were used (2.5 and 10%) which corresponded to 320 and 1280 shoots/m^2^, respectively.

The canopy model was placed 1 m from the paddle-type wave maker, and three different gap widths (GW) were considered: GW = h_v_/2, h_v_ and 2h_v_ (Fig 1B) together with experiments with no-gap (i.e. continuous canopy) and no-plants (i.e. bare sediment). All gaps started from x = 0 cm, the location of which was kept constant for all gaps widths (Fig 1B). Therefore, in all experiments the region situated from x<0 was covered by vegetation.

All measurements were taken in the central x–z plane, at y = 0. Eight vertical velocity profiles were measured at different x-positions (-1.5h_v_, -0.5h_v_, 0.25h_v_, 0.5h_v_, 1h_v_, 1.5h_v_, 2h_v_, 2.5h_v_) with a vertical separation of 2 cm. The measurements were made with an Acoustic Doppler Velocimeter (16 Mhz-ADV, Sontek Inc.). This instrument records (at 50 Hz) the three instantaneous velocity components at a single-point situated 5 cm from the probe tip with a sampling volume of 0.09 cm^3^. The ADV was placed in the flume in a downward looking configuration and connected to a PC with data acquisition software. The ADV instrument was configured to sample over 10-minute intervals (30,000 recordings per sampling interval).

The ADV was mounted in a frame and velocity profiles measured from z = 1 to 20 cm from the bottom of the flume, with a vertical resolution of 2 cm. Velocity measurements near the surface were limited by both wave shape and the 5-cm sampling volume of the ADV. To avoid spikes, beam correlations lower than 80% were removed. At two vertical positions (z = 8 cm and z = 20 cm above the bottom) low correlation was obtained. These ‘weak spots’ occurred when the first pulse emitted from the ADV was reflected at the bottom of the flume and met in time and space the second pulse within the sampling volume [5]. As the time lag between pulses depends on the velocity range, the ADV operational range was changed for these points (Sontek YSI, Acoustic Doppler Velocimeter Technical Instrumentation).

In order to obtain valid data acquisition within the canopy, a few stems were removed to ensure the ADV beam was not blocked and the acoustic receivers and transmitter performed properly [40,41]. To test the effect of the ‘hole’ on the ambient hydrodynamics, velocities were measured half a centimeter above the top of the canopy, both within and outside the hole. A 3% difference in velocities between ‘hole’ and ‘no hole’ canopies was observed at the highest SPF; only 1% difference was observed at the lowest SPF. We therefore concluded that the ‘hole’ made minimal modification to the ambient hydrodynamics.

### Method of analysis

In oscillatory flows the instantaneous velocity u can be decomposed into mean (U_c_), orbital velocity (U_w_) and turbulent velocity (u’) components as,

$$\text{u}={\text{U}}_{\text{c}}+{\text{U}}_{\text{w}}+{\text{u}}^{\prime }.$$ (1)

The above decomposition was made by using a phase-averaging technique [5,10]; the Hilbert transform was used to average oscillatory flow velocities with a common phase (φ). The velocity readings were binned into different phases as described by Pujol et al. [5]. The root mean square of u(φ) was then defined as the orbital velocity U_w,rms_ (hereafter denoted U_w_) as:

$${\text{U}}_{\text{w},\text{rms}}=\sqrt{\frac{1}{2\pi }\int_{0}^{2\pi }{\left(\text{u}\left(\varphi \right)-{\text{U}}_{\text{c}}\right)}^{2}\text{d}\varphi }.$$ (2)

To calculate the turbulent kinetic energy (TKE) profile for stationary velocity records, the instantaneous velocities (u, v, w) at each sampling point were decomposed into the sum of time-averaged velocities (U_c_, V_c_, W_c_), orbital velocities (U_w_, V_w_, W_w_) and the turbulent components (u’, v’, w’) as described in Eq (1); the TKE was calculated as:

$$\text{TKE}=1/2\left(\bar{{\text{u}}^{\prime 2}}+\bar{{\text{v}}^{\prime 2}}+\bar{{\text{w}}^{\prime 2}}\right).$$ (3)

## Results

**Fig 2:**
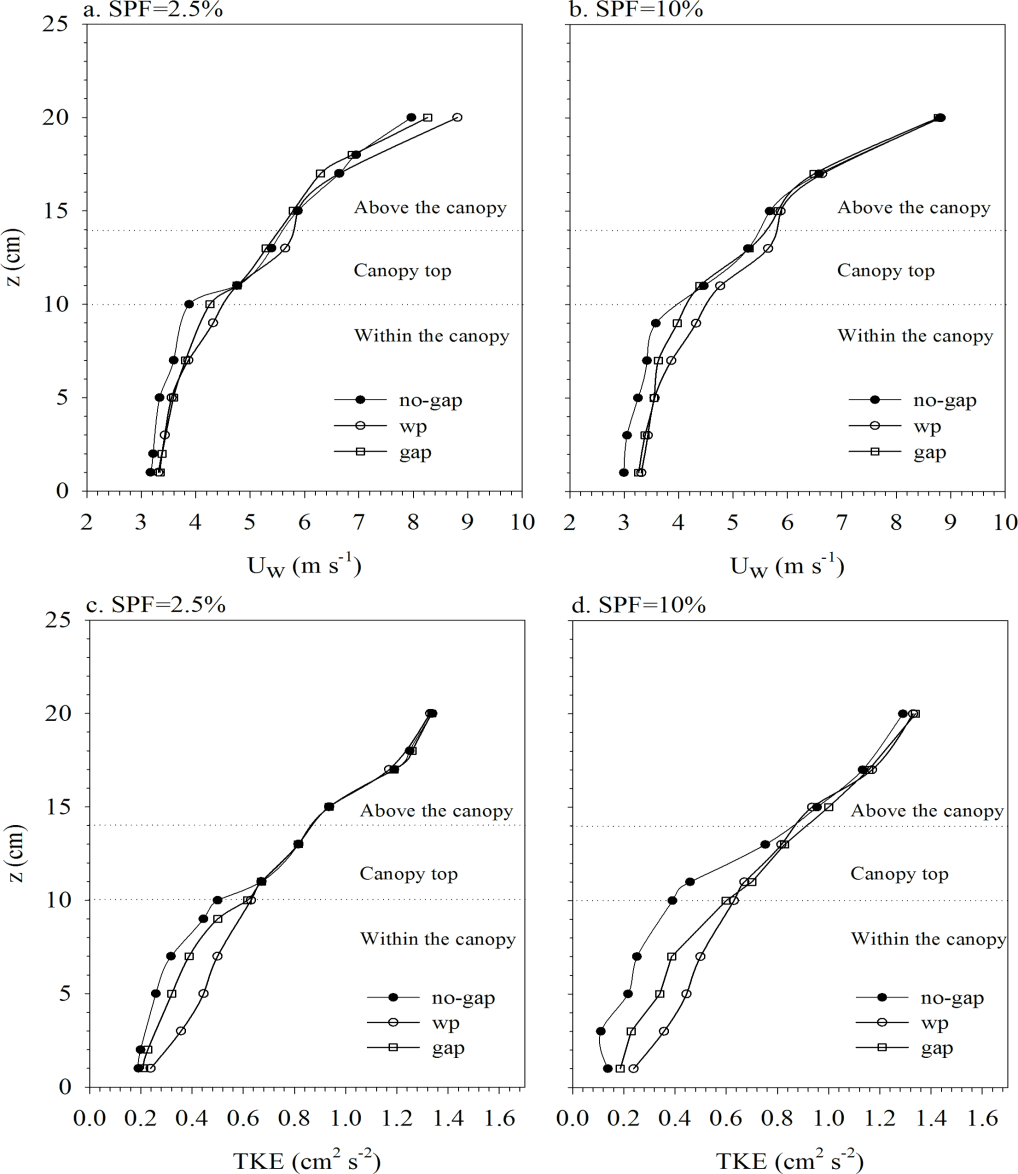
Vertical profiles of wave velocity (a and b) and turbulent kinetic energy (c and d) for the experiments with no-plants, no-gap and GW = 2hv. Left panels correspond to SPF 2.5% and right panels correspond to SPF 10%. The center of the gap was selected (x) for all in-gap wave velocity profiles. Horizontal dashed lines indicate the above-canopy layer, the canopy-top layer and the within-canopy layer.

The wave velocity profiles indicated that the water column can be divided into three layers: i) the above-canopy layer, ii) the canopy-top layer and iii) the within-canopy layer. In the within-canopy layer, the wave velocity in the no-gap experiments was lower than in the no-plants experiments, for both canopy densities of 2.5% (Fig 2A) and 10% (Fig 2B). In experiments with the largest gap (GW = 2h_v_), wave velocities in the center of the gap were larger than those obtained for the no-gap, for both SPF 2.5% (Fig 2A) and SPF 10% (Fig 2B). In fact, wave velocities within the gaps were close to those measured for the no-plants experiment. In the canopy-top layer, for the canopy density of 2.5% wave velocities for gapped canopies were close to those found for the no-plants experiment and those for the no-gap experiments (Fig 2A). However, for the canopy density of 10% (Fig 2B), wave velocities in this layer for both gapped and no-gap canopies were slightly lower than those in the no-plants experiment. In the above-canopy layer, wave velocities for the no-gap experiments were similar to those in the no-plants experiment and also similar to velocities within the gaps for the gapped canopies.

In the within-canopy layer of the no-gap experiments, the TKE was reduced compared to the TKE measured in this layer in the no-plants experiments. In the experiments with the largest gap (GW = 2h_v_), the TKE at the center of the gap was greater than that measured for the no-gap experiments but it remained below the TKE measured for the no-plants experiment. In the canopy-top layer for SPF 2.5%, the TKE for both gap and no-gap canopies remained close to the TKE measured in the no-plants experiments (Fig 2C). However, in the canopy-top layer for SPF 10% (Fig 2D), the TKE for the no-gap canopy was below the TKE for both gapped and no-plants experiments. In the above-canopy layer, the TKE for both gapped and no-gap canopies was close to the TKE measured in the no-plants experiment, for both canopy densities of 2.5% and 10%.

**Fig 3:**
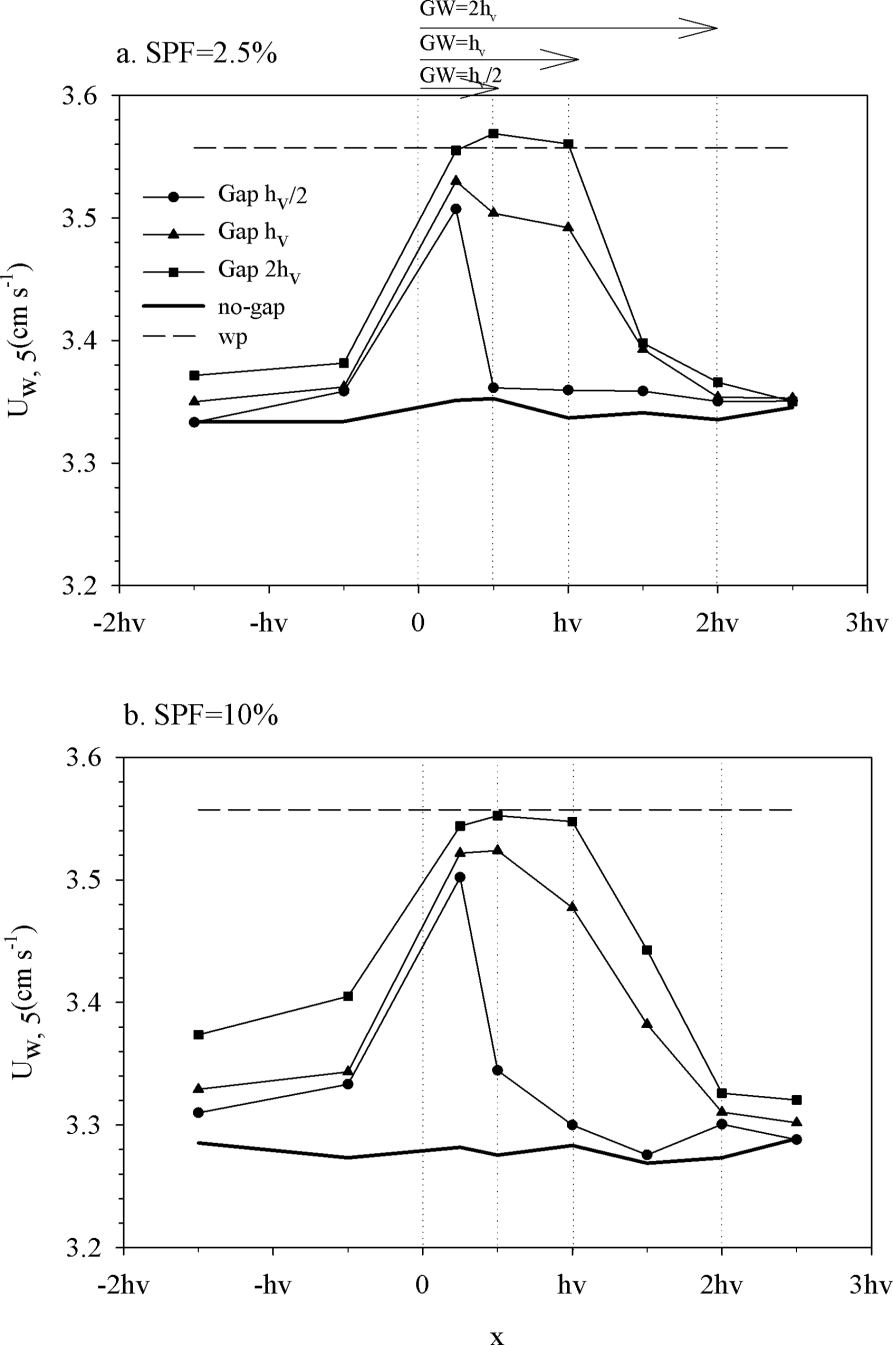
Wave velocity transect at z = 5 cm along the x-axis for the different experiments for a) SPF 2.5% and b) SPF 10%. Vertical dashed lines indicate the boundaries for the three different gap widths. Horizontal arrows at the top indicate the gap widths. The region situated at x<0 was covered by vegetation in all experiments.

To investigate the horizontal distribution of wave velocities along the gap and within the canopy, the wave velocity at z = 5 cm (U_w,5_) for the different gap widths was compared with the no-gap and the no-plants experiments (Fig 3). The x-axis corresponds to different points along the canopy and gap where measurements were taken (Fig 1). Wave velocities measured in the within-canopy layer approached the wave velocities for the no-gap canopy. In contrast, for the canopy with the largest gap (GW = 2h_v_)_,_ the wave velocity in this region was higher than that for the no-gap canopy for both canopy densities 2.5% (Fig 3A) and 10% (Fig 3B). In the within-gap layer, wave velocities gradually increased with x until the center of the gap, after which they decreased again, approaching the next boundary layer of the canopy. Once again, in the within-canopy layer, wave velocities were close to those measured in the no-gap canopies. The larger the gap, the greater the wave velocity within the gap. The maximum wave velocities measured within the gap adjacent to a canopy of SPF 2.5% (Fig 3A) were close to those measured within a gap adjacent to a canopy of SPF 10% (Fig 3B).

**Fig 4:**
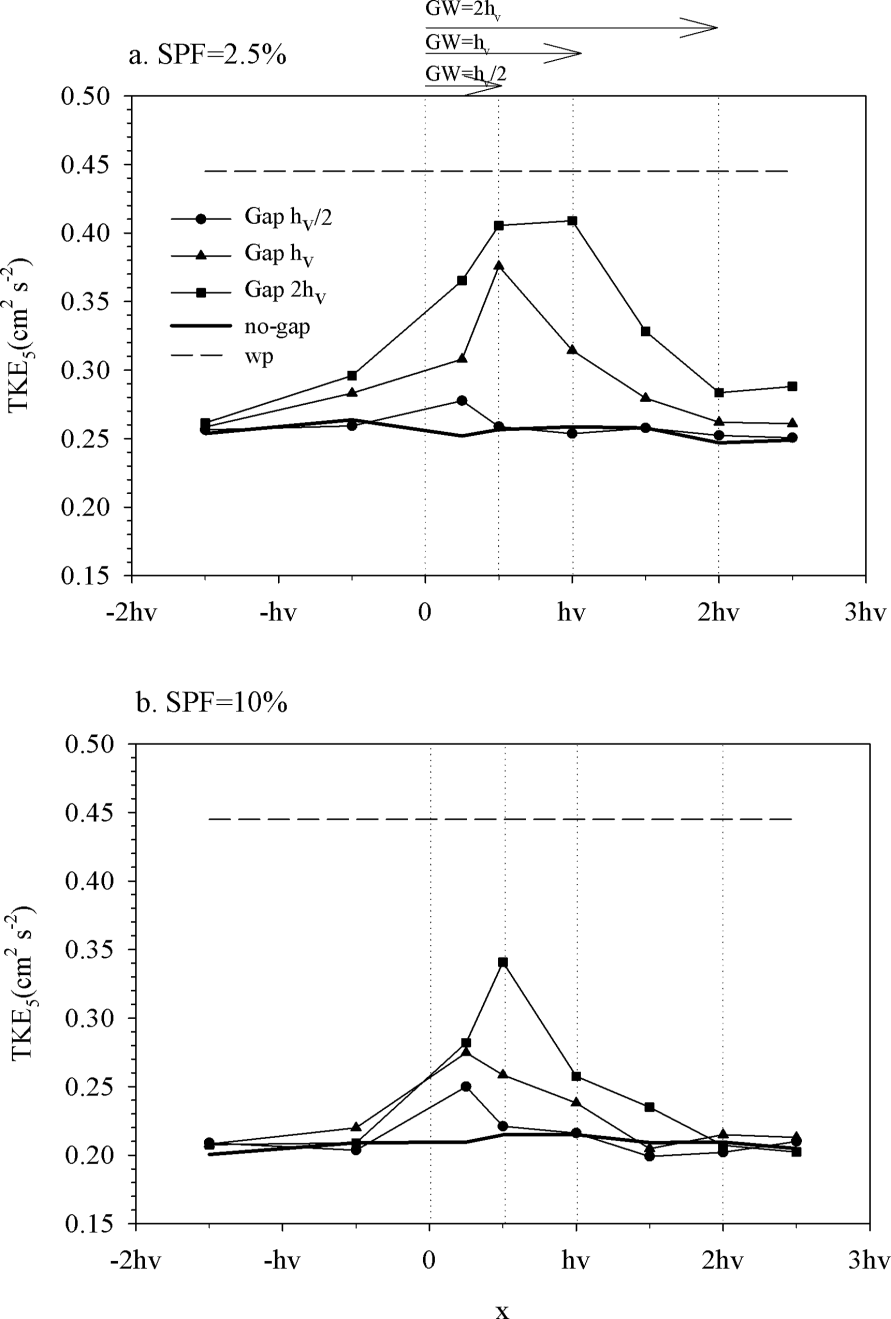
Turbulent kinetic energy transect at z = 5 cm along the x-axis for the different experiments for a) SPF 2.5% and b) SPF 10%. Vertical dashed lines indicate the boundaries for the three different gap widths. Horizontal arrows at the top indicate the gap widths. The region situated at x<0 was covered by vegetation in all experiments.

The TKE at z = 5 cm (TKE_5_) presented a similar pattern to the wave velocity. In the within-canopy layer, and for both canopy densities, TKE_5_ approached gradually the TKE_5_ found in the no-gap canopy (Fig 4). As found for the wave velocities, in the within-canopy layer the TKE_5_ gradually increased, reaching a maximum at the center of the gap, after which it decreased again as it approached the next canopy boundary layer. The maximum TKE_5_, measured within the gap for both SPF 2.5% (Fig 4A) and SPF 10% (Fig 4B) was lower than the TKE_5_ measured for the no-plants experiment. Note however that the maximum TKE_5_ measured within the gap for SPF 2.5% (Fig 4A) was greater than that measured within the gap for the SPF 10% (Fig 4B). Note also that TKE_5_ for the no-gap canopy with SPF 2.5% was a 25% greater than TKE_5_ measured for SPF 10% (Fig 4B). The TKE_5_ measured at the center of the gap decreased gradually with gap width.

As the TKE_5_ measured within the gap was found to be a function of the structural characteristics of the system (gap width and canopy density), a scaling law for TKE_5_ in the gap was developed. The parameters considered were the gap width (GW), the plant-to-plant distance (S), the orbital excursion length (A_w_ = U_w_/2πf) and the distance (x) from the nearest vegetation to the point within the gap where the TKE_5_ was measured.

**Fig 5:**
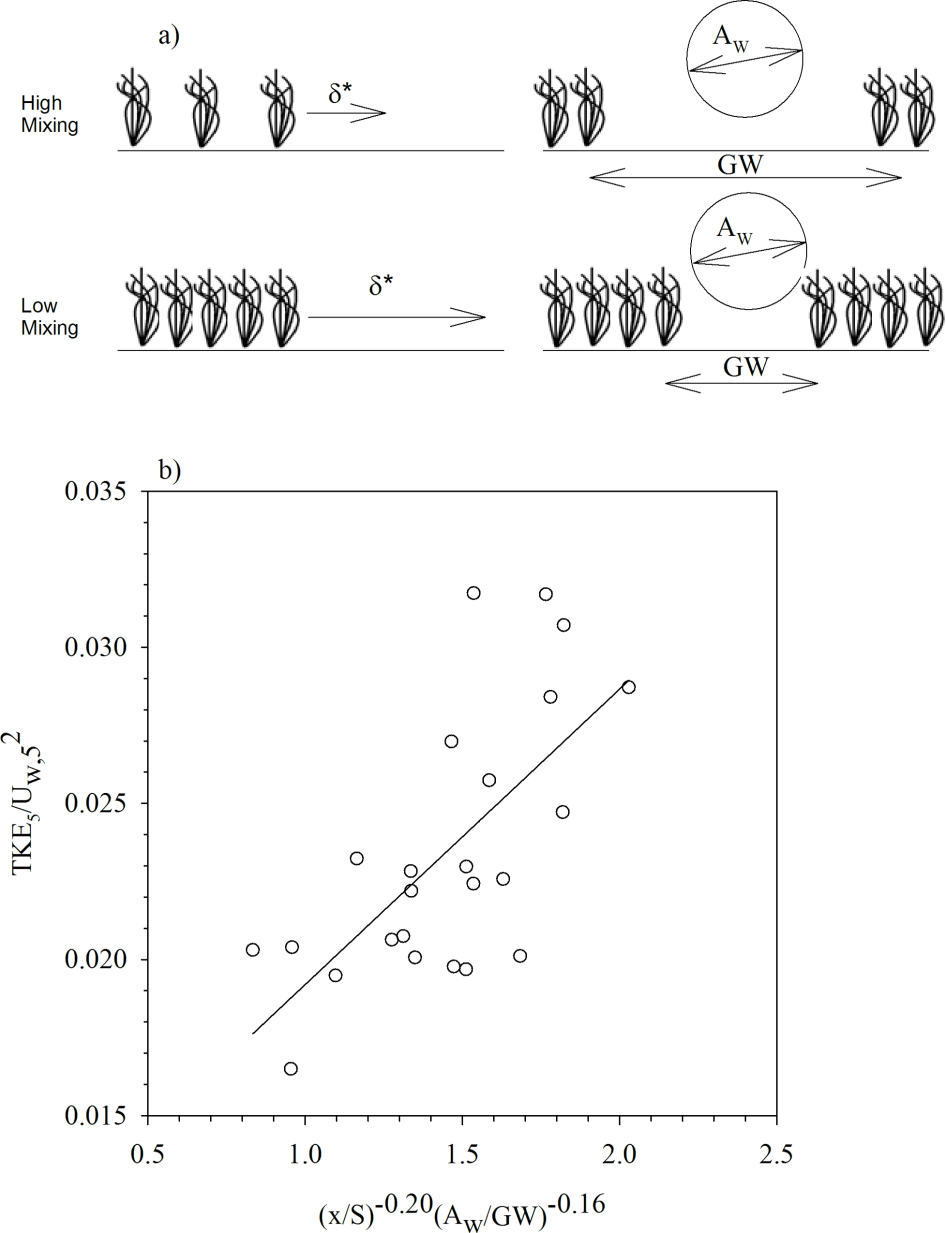
a) Schematic of the main length scales (δ* = x/S and A_w_/GW) used in the non-dimensional model. b) Relationship between the non-dimensional number (TKE/U_w_^2^)_5_ and (x/S)^α^(A_w_/GW)^β^. The distance x is the closest distance to the nearest canopy boundary. The fitting follows the equation ${\left(\frac{TKE}{U_{w}^{2}}\right)}_{5}=0.01{\left(\frac{x}{S}\right)}^{0.2}{\left(\frac{A_{w}}{GW}\right)}^{-0.16}+0.008$, with R^2^ = 0.7474, n = 24 and 99% of confidence.

The characteristic scales of the system were used to find a non-dimensional model that explains the TKE within a gap. The Buckingham pi-theorem was used to find the model, which is based on the assumption that physical laws should be independent of the units used to express the variables [42]. This theorem defines the relationship between the number of dependent variables (n) and the number of physical dimensions (m). The TKE at z = 5cm (TKE_5_) and U_w_ at z = 5 cm (U_w,5_) were considered proxies for TKE and U_w_ within the vegetation. In accordance with this scheme, we have 5 dependent variables (TKE_5_, GW, S, U_w,5_ and x); A_w_ should be a variable but as the wave frequency was not varied, it is only a function of U_w_, which is already a dependent variable. There are 2 dimensions (length and time) and therefore there will be 3 (n–m) non-dimensional parameters for this model (TKE_5_/U_w,5_^2^, x/S, A_w_/GW). The parameter TKE_5_/U_w,5_^2^ can be used to account for the mixing level associated with the wave. The parameter x/S (= δ*) can be used to characterize the sheltering at a distance x from a nearby vegetation with a certain plant-to-plant distance S (Fig 5A). Finally, the parameter A_w_/GW can be used to characterize the penetration of a wave with orbital excursion length scale A_w_ in a gap width GW (Fig 5A). The relationship between the governing variables used to quantify the turbulence within the gap will scale as:

$$\left(\frac{TKE_{5}}{U_{w,5}^{2}}\right)=K{\left(\frac{x}{S}\right)}^{\alpha }{\left(\frac{A_{w}}{GW}\right)}^{\beta },$$ (4)

where K is a non-dimensional constant and α and βare exponents.

Considering this equation, we plotted TKE_5_/U_w,5_ versus x/S for constant values of A_w_/GW (not shown) and found α to be -0.20. A plot of TKE_5_/U_w,5_^2^ versus A_w_/GW for constant values of x/S (not shown), found β to be -0.16. As such, the relationship between the TKE_5_/U_w,5_ and (x/S)^α^(A_w_/GW)^β^ presented a linear trend (Fig 5B). From the parameterization found in Eq (4) we can observe that the ratio (TKE_5_/U_w,5_^2^) increases with both S/x and GW/A_w_. Therefore, for a constant x/S, an increase in the gap width produces an increase in the ratio TKE_5_/U_w_^2^. This implies that there is a greater increase in the TKE_5_ than the U_w,5_ for gapped canopies when compared to a no-gap canopy. Furthermore, from Eq (4) and given that S decreases with increasing canopy density, we found that TKE_5_ in the centre of a gap adjacent to sparse canopies is greater than found adjacent to dense canopies. In other words, sparse gapped canopies have low sheltering capability. This expected result provides confidence in the scaling arguments.

**Fig 6:**
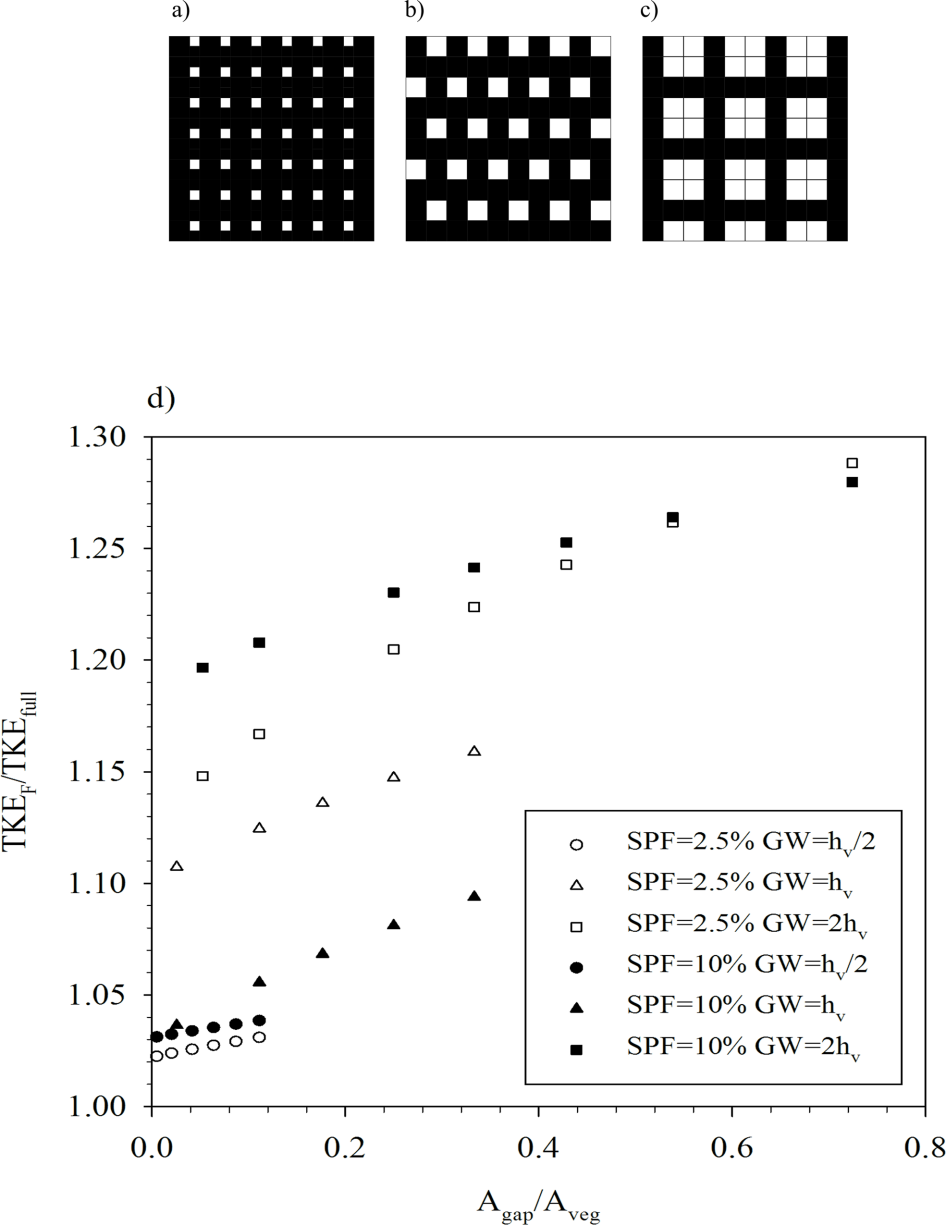
Schematic of the maximum canopy fragmentation for a) GW = h_v_/2, b) GW = h_v_ and c) GW = 2h_v_. The canopy used in the schematic is 10h_v_×10h_v_. Vegetated areas are in black and gaps are in white. d) Relationship between TKE_F_/TKE_full_ and A_gap_/A_veg_ for the SPF 2.5% (open symbols) and SPF 10% (solid symbols) and for the three gap widths GW = h_v_/2, GW = h_v_ and GW = 2h_v_.

In the field, we would expect a fragmented canopy to contain one or more gaps. A simple fragmentation canopy model was developed, based on our results, to evaluate the degree of turbulent mixing within a fragmented canopy compared to the case of a canopy without gaps. The model considered a canopy of finite dimensions and gaps of dimensions h_v_/2xh_v_/2, h_v_xh_v_ and 2h_v_x2h_v_ were implemented within the canopy. The maximum number of gaps within the canopy was limited by a minimum canopy patch (i.e. the distance between gaps), which was given as h_v_ in both x- and y-direction. This minimum patch width imposed a maximum number of gaps within the canopy that was different for each gap width considered. In Fig 6A–6C the maximum gaps for gap widths of h_v_/2, h_v_ and 2h_v_ are presented for a canopy of 10h_v_x10h_v_. The fragmentation model considered here was for a canopy of 100h_v_x100h_v_, i.e. 100 times that shown in Fig 6A–6C. Therefore, the maximum ratio A_gap_/A_veg_ was 0.11, 0.33 and 0.72 for GW h_v_/2, h_v_ and 2h_v_, respectively. In the model, different levels of fragmentation for each gap width were considered with ratios A_gap_/A_veg_ below the maxima indicated above. The total area of gaps (A_gap_) and vegetation (A_veg_) were calculated for each gap width (Table 1). The first data row in Table 1 shows the maximum possible gap area, and the minimum vegetated area, for each gap width. The subsequent rows show fragmented canopies with lower total gap areas. Using the data presented in Fig 4, the TKE of the fragmented canopy (TKE_F_) was calculated using a weighted mean of the within-canopy TKE and the TKE in different sized gaps as:

$${\text{TKE}}_{F}=\left({\text{A}}_{\text{gap}}\times {\text{TKE}}_{gap}+{\text{A}}_{veg}\times {\text{TKE}}_{veg}\right)/\left({\text{A}}_{\text{gap}}+{\text{A}}_{veg}\right)$$ (5)

**Table 1:** Total gap area and total vegetation area in the canopy, for the three different gap widths.

GW = h_v_/2		GW = h_v_		GW = 2h_v_	
A_gap_(%)	A_veg_(%)	A_gap_(%)	A_veg_(%)	A_gap_(%)	A_veg_(%)
10	90	25	75	42	58
8	92	20	80	35	65
6	94	15	85	30	70
4	96	10	90	25	75
2	98	2.5	97.5	20	80
0.5	99.5			10	90

The ratio between the TKE_F_ and the TKE_full_ (i.e. the TKE in the no-gap case) was plotted as a function of A_gap_/A_veg_ for SPF 2.5% and 10% canopies (Fig 6D). The larger the ratio of the area of the gaps to the area of vegetation (A_gap_/A_veg_), the greater the mixing in the fragmented canopy (TKE_F_/TKE_full_). Furthermore, for the same ratio A_gap_/A_veg_, the mixing will be greater in canopies with large gaps than in canopies with small gaps, despite having the same total gap area (A_gap_). For the gap widths of h_v_/2 and h_v_, the mixing was lower for the dense canopy than for the sparse canopy. However, the presence of large gaps of width 2h_v_ destabilized a dense canopy of 10% SPF more than a sparse canopy of 2.5% SPF.

## Discussion

### Interactions between the canopy, a transversal gap and local hydrodynamics

In a transversal gap with its main axis perpendicular to the wave direction, the vertical attenuation of wave velocities and the turbulent kinetic energy within the gap depended on both the gap width and the canopy density.

For gaps widths less than twice the canopy height (GW<2h_v_), the presence of the nearby canopy sheltered the gap, reducing the wave velocity and the TKE within it. The canopy density of the adjacent vegetation was the more important parameter determining the TKE sheltering within the gap. In all the experiments, the orbital excursion length scale (A_w_) was smaller than the gap width (GW). The greatest ratio A_w_/GW was 0.24, when waves always entered the gap irrespective of its width, [9].

Both the TKE_5_ and the wave velocity within the gapped canopy, but close to the gap-vegetation interface, were greater than those measured in no-gap canopies, showing the effect of the wave penetration into the canopy due to the presence of the adjacent gap. This impact of gaps on canopy hydrodynamics must be taken into account when making extrapolations from experimental results to the field environment, where canopy gaps are common.

The total kinetic energy measured within the submerged canopy was found to be 10%-25% of that measured above the canopy, in agreement with previous work [8] while the total kinetic energy adjacent to bare sediments far from canopies was found to only be reduced to 50%. Our data has quantified that the canopies provide up to 40% more sheltering to adjacent sediments, and that the degree of sheltering changes across the vegetation–gap interface. These results agree with previous work using a canopy density of 200–400 shoots/m^2^, comparable to the SPF 2.5% used in this study [8]. Granata et al. [8] also found that the total kinetic energy in regions without plants but close to a canopy was lower than that measured over bare sediments far from the canopy boundary, again highlighting the role of the canopy in sheltering the adjacent bare soil.

We have shown that the hydrodynamic environment within gaps and within vegetation close to gaps, may therefore vary strongly depending on gap width and plant density. Ecological benefits of gaps (e.g. access to pelagic organisms, import of pelagic seeds into a canopy) will be a function of these hydrodynamics, and raises the possibility of canopies optimizing their ecological benefit by modification of canopy structural characteristics. Such ecological optimization of the hydrodynamic environment has recently been proposed for rigid Rangeomorph communities, which were non-motile organisms that inhabited the deep ocean benthic boundary layer until 542 million years ago [43]. Ghisalberti et al. [43] demonstrated that there was a selective advantage for tall individuals related to access to the overlying flow. They suggested that higher flows across the surfaces of tall communities permitted more rapid uptake of nutrients, than for short communities.

The non-dimensional model of Eq (4) proposed that the hydrodynamics varied as a function of two non-dimensional parameters. The first parameter, x/S, represents a non-dimensional boundary layer length scale δ*; this is the distance over which the canopy shelters the gap. In the scheme shown in Fig 5A, large δ* (through small S) implies a low TKE/U_w_^2^, i.e. a low mixing due to the sheltering by the nearby vegetation. The second scale, A_w_/GW, is a measure of the wave penetration within the gap, as it compares the orbital excursion length scale of the wave with the gap width (Fig 5A). Large A_w_/GW indicates a low penetration, i.e. a decrease in the mixing level (i.e. a decrease in TKE/U_w_^2^) within the gap. Given the relationship developed through the non-dimensional analysis, we arrive at Eq (6) to describe the turbulent kinetic energy within the gap as a function of easily measured variables:

$$TKE_{5}=\left[0.01{\left(\frac{x}{S}\right)}^{0.2}{\left(\frac{A_{W}}{GW}\right)}^{-0.16}+0.008\right]U_{w,5}^{2}$$ (6)

This equation can be used to explore the effects of sheltering on particle and nutrient fluxes into and out of the canopy.

Koch and Gust [35] used a modified Richardson number to estimate the stability of the water parcels above and within a meadow. The modified Richardson number was defined as the ratio of the stabilizing forces (difference in canopy density between two water parcels) to the destabilizing forces (velocity gradient between water parcels). We explored the use of this formulation to characterize a canopy with gaps, following:

$$Ri=\frac{g\frac{d\rho }{dz}}{\rho {\left(\frac{dU}{dz}\right)}^{2}}$$ (7)

where g is the acceleration of gravity, dρ/dz is the change in the seagrass density, i.e. the change in SPF between the two water depths considered, and dU/dz is the change in velocity over depth. A decrease in the Richardson number would therefore indicate an increase in the mixing rate between water parcels, i.e. stability is decreased. Our results show that an increase in canopy density (i.e. a high dρ/dz) resulted in an increase in dU/dz due to wave velocity attenuation by the vegetation. This wave velocity attenuation suggests an increase in the stability in the within-canopy layer. However, Eq (7) shows that an increase in dU/dz should decrease Ri, i.e. instability is increased, which is opposite to what was initially expected. Due to this fact, the Richardson number as formulated in Eq (7) was not considered a good indicator to evaluate the degree of mixing over a gapped canopy. Instead, the ratio between the TKE of a canopy with a gap and the TKE for a canopy without a gap (both at z = 5cm) was considered the best parameter to account for the degree of instability within the canopy due to the presence of gaps.

### Hydrodynamics of a fragmented canopy

The level of mixing within a fragmented canopy was revealed by the developed model. Of particular interest is the finding that for the same total gap area (A_gap_), a fragmented canopy with larger gaps resulted in a higher level of mixing than with smaller gaps. Therefore, the stability of a fragmented canopy is greater with a larger number of small gaps than with a few large gaps (Fig 6). In addition, a larger gap area in a dense fragmented canopy is needed to reach the same mixing level than that experienced by a sparse canopy. This result quantifies the sheltering effect of the nearby canopy on the adjacent gap. All the curves in Fig 6 plateau as A_gap_/A_veg_ becomes large, approaching the value expected in a completely fragmented canopy (i.e. for TKE_F_ = TKE_gap_). This plateau can be attributed to the imposed limit of the smallest possible patch size (h_v_). The model results may explain the finding that lower nutrient concentrations were found in small patches of vegetation within a fragmented canopy [33] than in continuous meadows. The nutrients in small patches may be flushed out of the patch due to the increased mixing between the patch and the nearby gap. This predicted increase in the mixing level in fragmented canopies also supports the observed increased diffusion coefficient in fragmented canopies under unidirectional flows [44]. Modifications in the mixing and turbulent processes in gapped canopies may also result in changes in the water turbidity, which in turn may reduce light intensity and the consequent loss of seagrass areas [45]. Therefore, as claimed by Recio et al. [46], the hydromorphological regime may be an indicator of the canopy status, and increased fragmentation of canopies (whether of natural or anthropogenic origin) may significantly alter local hydrodynamics and sedimentation patterns.

As discussed by Duarte [47], seagrasses are increasingly fragmented and they are in significant global decline. Although all seagrass canopies have edges, an increase in the level of fragmentation results in an increase in the length of edges relative to the vegetated area. It is known that the ecological diversity and habitat of edges may differ from those of interior habitats [48]. Therefore, those species requiring interior habitats may respond negatively to habitat fragmentation. Macreadie et al. [49] found an increase in fish density in the vegetated areas of a fragmented canopy compared with the fish density in continuous canopies. This fact imposes a limit for the small remaining patches to withstand the pressure of herbivores. Gera et al. [33] pointed out that the structural reduction of the seagrass *Posidonia oceanica* due to the combined effect of fragmentation and herbivory might compromise the seagrass functional role. The comparative effects on canopy ecology of edge length versus gap width have not been quantified. The results presented here characterize the hydrodynamic changes when the gap area increases versus the vegetated area, shifting the habitat distribution that in turn affects the canopy ecology.

Faunal abundance at the edges of a canopy can be related to the amount of detrital material settled in this region [48]. Bologna and Heck [50] suggested that transported larvae encountering the seagrass structure settle due to the flow reduction and become concentrated at edges, which may explain the high abundance of some sessile species at the edges of seagrasses. In the present study, the wave velocity and the turbulent kinetic energy were reduced within a canopy at a distance of one plant height from the edge of the canopy. This is expected to be the region with a greater deposition of allochthonous material. Bologna and Heck [51] found a greater abundance of scallops at the edges of a *Thalassia Testudinum* bed compared to the interior. They attributed this finding to a greater flux of food at the edges, coinciding with higher scallop growth rates but also with a higher predation potential. Ricart et al. [2] demonstrated that fragmented canopies influenced the exchange of materials, with a decrease in detrital seagrass leaves in patchy meadows. Our study has quantified the extent to which sheltering is reduced in fragmented canopies which may explain the reduced amount of leaf detritus observed by Ricart et al. (2015).

Reusch and Chapman [52] demonstrated that the presence of eelgrass meadows provides habitat to mussels through the protection from waves and currents. However, storms may cause uprooting of meadows and promote mussel dislodgement. Our results demonstrated that a storm would produce greater mixing in fragmented canopies compared to non-fragmented canopies, resulting in a greater hydrodynamic stress for the communities sheltered by the canopy. However, Reusch and Williams [53] found less abundance of the non-native mussel *Musculista senhouisa* inside a *Zostera marina* meadow than on the unvegetated sediment. They suggested that the mussel received less food inside the meadow compared to the bare sediment. In such cases, an increase in fragmentation could be favorable for the establishment of this invasive species. Vinther et al. [54] and Williams [55], noted that the ecological effect of invasive species on seagrass and associated communities have been predominantly negative.

The results presented here quantify the change in mixing within a canopy affected by gaps. Gaps have more effect on sparse canopies than dense canopies. Our results can be used to quantify the threshold level of canopy fragmentation above which the canopy becomes unstable. This threshold is a function of canopy density and therefore anything that impacts shoot density will ultimately affect the resilience of the canopy to hydrodynamic stress. For example Marbà and Duarte [56] found that an increase in water temperature triggered a decrease in the shoot density, highlighting the potential impact of climate change. The combined effects of increased water temperatures and canopy fragmentation induced by human activities, will likely make those meadows more vulnerable. In such cases, sediments will be exposed to high mixing events, reducing the carbon storage capacity of the canopy through sediment resuspension. Marbà and Duarte [56] also showed that the canopy shoot density decreased with water depth. Our model predicts that when wave velocities approach those that plants can withstand, a fragmented canopy in deeper water will be more vulnerable than a fragmented canopy in shallow water [57]. This has implications for the management of human activities that might trigger the fragmentation of canopies (e.g. boat moorings).

## Conclusions

These results have quantified the complex interactions between canopy density, the proportion of canopy that is fragmented, gap width, and the sheltering experienced within the gap. The presence of a single transversal gap within a canopy increased both the wave velocity and the turbulent kinetic energy within the gap, compared to what is found within a continuous canopy (without a gap). A large gap within a canopy produced an increase in the turbulent kinetic energy and therefore a reduction in the sheltering offered by the vegetation. The wave velocity within small gaps was attenuated more than within large gaps. However while aquatic vegetation attenuates waves and currents, fragmented canopies are likely to experience a loss of sheltering. In terms of TKE, fragmented canopies with small gap widths are able to produce more sheltering than fragmented canopies with larger gaps despite the total gap area being equal. Furthermore, the canopy density plays an important role in sheltering the nearby gap from turbulent kinetic energy. Gaps within a sparse canopy will experience less sheltering; this effect is a function of gap width. For larger gaps in sparse canopies, both wave velocities and turbulent kinetic energies may be closer to those found over bare sediments.

All of these relationships will impact the fluxes of biological particles, nutrients and sediments between the canopy and the adjacent zones, and therefore the canopy ecological function. Our results raise the question whether canopies may optimize their structural characteristics, particularly plant density, to moderate the impacts of gaps and fragmentation and optimize ecological function, however we have shown that plant density interacts with gap width and degree of fragmentation to facilitate sheltering in a manner not previously predicted.
